# Development of a Tissue-Engineering Vascular Graft for Use in Congenital Heart Surgery

**DOI:** 10.1016/j.ebiom.2014.10.001

**Published:** 2014-10-05

**Authors:** Toshiharu Shinoka

**Affiliations:** Department of Cardiothoracic Surgery, The Heart Center, Nationwide Children's Hospital, United States; Department of Surgery, Ohio State University, United States

Congenital cardiac anomalies represent the most common birth defect affecting nearly 1% of all live-births. Severe forms of congenital heart disease are life-threatening and require surgical intervention. Despite significant advances in the surgical and medical management of this disease, it remains a leading cause of death in the newborn period and lifelong morbidity for survivors. One significant source of morbidity and mortality arises from complications associated with the use of currently available prosthetic vascular grafts, patches, and replacement heart valves that are frequently needed to perform reconstructive procedures. These risks include a greater chance of thromboembolic complications, poor durability due to neointimal hyperplasia, increased susceptibility to infection, and lack of growth capacity. The development of better biomaterials with growth potential could substantially improve the outcomes of children requiring congenital heart surgery by reducing the number of graft-related complications and enabling earlier definitive surgical repair without risk of serial re-operation.

Tissue engineering offers an innovative solution to this significant problem. Using tissue engineering methods, bioprosthetics can be made from an individual's own cells creating a living material with excellent biocompatibility and the ability to grow, repair, and remodel. One promising approach utilized by several research groups is the use of a biodegradable scaffold seeded with autologous cells. In 2001, our group pioneered the development and translation of tissue-engineering technologies to create vascular grafts for use in the surgical repair of congenital cardiac anomalies, using a biodegradable polymer conduit (Shinoka et al., 2001, Hibino et al., 2010). de Mel and colleagues used a nanocomposite biomaterial-based small diameter vascular graft and induced endothelialization *in situ* (de Mel et al., 2009). Thomas and Nair demonstrated an electrospinning technique to generate biodegradable tubular scaffolds for seeding vascular smooth muscle cells (Thomas and Nair, 2013). Our group is currently performing the first FDA-approved clinical trial evaluating the use of tissue engineered vascular grafts (TEVG) (IDE 14127). The TEVG is made by seeding bone marrow-derived mononuclear cells onto a biodegradable tubular scaffold.

In 2012, Olausson and colleagues described a novel and unique attempt to develop tissue-engineered vein using decellularized allogenic vein as the scaffold, and autologous endothelial and smooth muscle cells differentiated from cultured mesenchymal stem cells obtained from 20 mL of bone marrow, although the graft was narrowed at 1 year after the surgery and the patient required the second application of new graft implantation (Olausson et al., 2012). Now in *EBioMedicine*, they describe another clinical application of bioengineered vascular graft using an autologous peripheral whole blood instead of bone marrow cells, although one graft was also explanted 7 months after initial surgery due to the narrowing. It is indeed very challenging for any type of vascular conduit to maintain the good patency in low-flow venous system. In fact, our previous clinical results also demonstrated that 4 out of 25 venous conduits required balloon angioplasty due to narrowing in the long-term periods (Hibino et al., 2010).

Many readers would be very interested in the histology of explanted tissue in the patient and would like to know the fate of seeded cells. We have experience of using cultured endothelial cells for seeding our graft. It was difficult to maintain high quality-controlled production process in a stable condition, especially for cell expansion of endothelial cells *in vitro*. Despite the number of preparatory steps needed for the authors' graft, the rewards look small. If the decellularized vein is easily available, I would recommend trying to use bone marrow mononuclear cells expressing a cytokine effect to recruit host-derived endothelial cells and smooth muscle cells onto the vein, so that the time-consuming cell expansion step can be eliminated. In fact, we demonstrated that the seeded mononuclear cells in the vascular grafts were eventually replaced by cells from adjacent vessels, probably via the body's innate healing mechanism, resulting in neovessels of entirely host cell origin (Hibino et al., 2011).

Finally, I would like to congratulate the authors on this successful, compassionate clinical application in these very difficult patients for whom conventional approaches were not feasible or hazardous.

## Disclosure

Dr. Shinoka reports grants from Gunze Ltd.

## References

[bb0015] de Mel A., Punshon G., Ramesh B. (2009). *In situ* endothelialization potential of a biofunctionalised nanocomposite biomaterial-based small diameter bypass graft. Biomed. Mater. Eng..

[bb0010] Hibino N., McGillicuddy E., Matsumura G. (2010). Late-term results of tissue-engineered vascular grafts in humans. J. Thorac. Cardiovasc. Surg..

[bb0030] Hibino N., Villalona G., Pietris N. (2011). Tissue-engineered vascular grafts form neovessels that arise from regeneration of the adjacent blood vessel. FASEB J..

[bb0025] Olausson M., Patil P.B., Kuna V.K., Chougule P., Hernandez N., Methe K., Kullberg-Lindh C., Borg H., Ejnell H., Sumitran-Holgersson S. (2012). Transplantation of an allogenic vein bioengineered with autologous stem cells: a proof-of-concept study. Lancet.

[bb0005] Shinoka T., Imai Y., Ikada Y. (2001). Transplantation of a tissue-engineered pulmonary artery. N. Engl. J. Med..

[bb0020] Thomas L.V., Nair P.D. (2013). The effect of pulsatile loading and scaffold structure for the generation of a medial equivalent tissue engineered vascular graft. Biores. Open Access.

